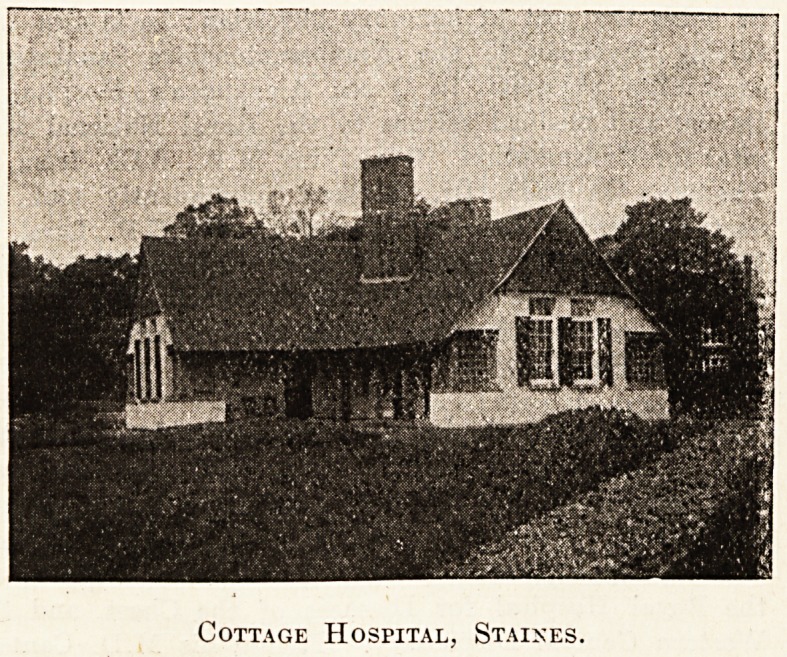# Cottage Hospital, Staines

**Published:** 1914-09-26

**Authors:** 


					September 26, 1914. THE HOSPITAL 701
HOSPITAL ARCHITECTURE AND CONSTRUCTION.
Cottage Hospital, Staines,
The plans which we publish appear to have been
successful in winning a competition, but how the
competition was decided or whether any skilled
advice was obtained we are not aware.
The plans are in some respects skilfully designed,
but it is evident that the.limited sum (?1,250, to
include entrance drive and gates) has bad the effect
of restricting the accommodation.
The wards are planned with their axes at an angle
of 45 to the axis of the main building, which
gives great advantages in the matter of aspect and
ventilation. To each ward is provided a w.c.,
which is approached by a long passage, or " inner
hall," with cross-ventilation. No provision beyond
an ordinary sink appears to be made for emptying
or cleansing bed-pans. Moreover, these two w.c.s
are the only ones provided, so that the staff must
apparently use one or the other?a very objection-
able arrangement.
The kitchen is in front, close to the entrance,
with the scullery adjoining. This does not seem
a very happy piece of planning. It would have
been better to have put the operation room, which
in so small a hospital as this could also have been
used as a surgery, in front and placed the kitchen
offices within easier distance of the wards.
Provision is made for one nurse and a probationer
and for servants.
The architect was Mr. Leslie Moore, A.R.I.B.A.
COTTAGE. HOSPITAL STAINES ?
MAIM EttTftflNGE
6R0UHDFLOOR PLAN
NOTt
WflrtDS IBVlHHEIQHT
WOUND VIV FLOORS Of
.IDMintSTMTION d'6'
LESLIE MOOKC. n-R-lt-H
3 RftYMOHD BUILDItiqS
C/fflYJ liin LOHDOM W.C
FIRST FLOOR PLflH
Cottage Hospital, Staines,

				

## Figures and Tables

**Figure f1:**
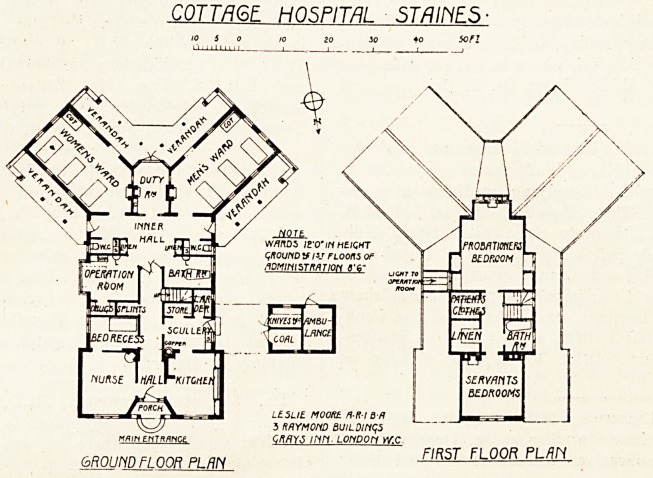


**Figure f2:**